# Methods for assessment of patient adherence to removable orthoses used after surgery or trauma to the appendicular skeleton: a systematic review

**DOI:** 10.1186/s13063-020-04456-2

**Published:** 2020-06-08

**Authors:** Gareth Davies, Daniel Yeomans, Zoe Tolkien, Irene A. Kreis, Shelley Potter, Matthew D. Gardiner, Abhilash Jain, James Henderson, Jane M. Blazeby

**Affiliations:** 1 Bristol Centre for Surgical Research, Population Health Sciences, Bristol Medical School, 39 Whatley Road, Clifton, Bristol, BS8 2PS UK; 2 Department of Trauma and Orthopaedic Surgery, North Bristol NHS Trust, Southmead Road, Bristol, UK; 3 Clinical Effectiveness Unit, Royal College of Surgeons England, London, UK; 4 Nuffield Department of Orthopaedics, Rheumatology and Musculoskeletal Sciences, University of Oxford, Nuffield Orthopaedic Centre, Windmill Road, Headington, Oxford, OX3 7HE UK; 5 Department of Plastic Surgery, Frimley Health NHS Foundation Trust, Camberley, GU16 7UJ UK; 6 Department of Plastic Surgery, Imperial College London Healthcare NHS Trust, London, SW7 2AZ UK; 7 Department of Plastic Surgery, North Bristol NHS Trust, Southmead Road, Bristol, UK

**Keywords:** Systematic review, Adherence, Orthoses, Appendicular skeleton, Orthopaedics

## Abstract

**Background:**

Patient adherence to treatment is a key determinant of outcome for healthcare interventions. Whilst non-adherence has been well evidenced in settings such as drug therapy, information regarding patient adherence to orthoses, particularly in the acute setting, is lacking. The aim of this systematic review was to identify, summarise, and critically appraise reported methods for assessing adherence to removable orthoses in adults following acute injury or surgery.

**Methods:**

Comprehensive searches of the Ovid versions of MEDLINE, Embase, AMED, CINAHL, Central, the Cochrane Database of Systematic Reviews, and SPORTDiscus identified complete papers published in English between 1990 and September 2018 reporting measurement of adherence to orthoses in adults following surgery or trauma to the appendicular skeleton. Only primary studies with reference to adherence in the title/abstract were included to maintain the focus of the review. Data extraction included study design, sample size, study population, orthosis studied, and instructions for use. Details of methods for assessing adherence were extracted, including instrument/method used, frequency of completion, number of items (if applicable), and score (if any) used to evaluate adherence overall. Validity and reliability of identified methods were assessed together with any conclusions drawn between adherence and outcomes in the study.

**Results:**

Seventeen papers (5 randomised trials, 10 cohort studies, and 2 case series) were included covering upper (*n* = 13) and lower (*n* = 4) limb conditions. A variety of methods for assessing adherence were identified, including questionnaires (*n* = 10) with single (*n* = 3) or multiple items (*n* = 7), home diaries (*n* = 4), and discussions with the patient (*n* = 3). There was no consistency in the target behaviour assessed or in the timing or frequency of assessment or the scoring systems used. None of the measures was validated for use in the target population.

**Conclusions:**

Measurement and reporting of adherence to orthosis use is currently inconsistent. Further research is required to develop a measurement tool that provides a rigorous and reproducible assessment of adherence in this acute population.

**Trial registration:**

PROSPERO: CRD42016048462. Registered on 17/10/2016.

## Background

Adherence to treatment, defined by the World Health Organization (WHO) as ’the extent to which a person’s behaviour corresponds with agreed recommendations from a health care provider’ [1], is a key determinant of healthcare outcomes. Within a clinical setting, accurate documentation of adherence aids a clinician’s judgement of whether poor adherence or ineffectiveness of a prescribed intervention may be responsible for a patient’s clinical progress. Similarly, within a research setting, measuring adherence to a specific intervention is crucial in accurately determining its efficacy and in understanding how this may affect its effectiveness in clinical practice [2]. Even the most efficacious interventions are only as effective as the degree to which patients comply with the recommendations of healthcare professionals [3, 4]. For this reason, the WHO have identified improving patient adherence as one of their key research priorities [1].

Reasons for non-adherence to medical guidance are multi-faceted, with socioeconomic, health-care system-related, condition-related, treatment-related, and patient-related factors all contributing [1]. With regard to orthosis use, specific factors such as discomfort, ill fit, inconvenience, skin irritation, and issues such as disturbed sleep have all been postulated as reasons for non-adherence [5–8]. Although not a straightforward relationship, there is evidence to support that adherence to the prescribed use of an orthosis in acute conditions leads to superior outcomes following muscle tears [9] and tendon injuries [10–12] and post-operatively [13].

Measuring adherence is a complex task. There are multiple definitions of the term, it can fluctuate over time, and simply measuring adherence can influence the behaviour itself [14]. Accurately and reliably measuring adherence becomes more difficult with interventions that rely on the co-operation and engagement of patients away from direct supervision of healthcare professionals. The use of removable orthoses is an ideal example of this, as these devices are often prescribed with complex treatment protocols to be followed at home.

Systematic reviews have previously described and appraised the measurement tools used for assessing adherence in home-based exercise programmes [15–17] and in medication adherence [18], but to our knowledge none exist in patients prescribed removable orthoses.

The aim of this review was to identify, summarise, and critically appraise the methods reported in the literature for assessing patient adherence to treatment with a removable orthosis in adults following surgery or trauma to the appendicular skeleton. This review formed part of the MALIT (Mallet Injury Trial) study, preliminary work to inform the design of a future randomised controlled trial (RCT) in mallet injury splinting, to identify candidate measures of adherence for use in a future trial.

## Methods

The protocol for this systematic review was registered on the PROSPERO International Prospective Register of Systematic Reviews (reference number CRD42016048462) prior to commencing data extraction.

### Literature search strategy

The Ovid search platform (OvidSP) versions of MEDLINE, Embase, AMED, Cumulative Index to Nursing and Allied Health Literature (CINAHL), Central, the Cochrane Database of Systematic Reviews, and SPORTDiscus were searched using a search strategy developed by members of the team in consultation with a research librarian trained in healthcare research methodology. The search strategy included terms for ‘orthoses’ combined using the operator OR including ‘splints/; splint* in title/abstract; braces/; brace* in title/abstract; orthopaedic equipment/; orthotic devices/; orthos#s in title/abstract; orthotic in title/abstract; ‘appendicular skeleton’ (hand, arm, leg, finger) and ‘injury’ combined using the operator ‘AND’. Details of the search strategy are included in Additional file 1.

The search was limited to human studies, in adults, published in English from 1990 to September 2018. Studies published prior to 1990 were excluded, as they were unlikely to reflect current practice. Abstracts and conference reports were not included due to difficulties evaluating incomplete information. Systematic reviews and qualitative research were excluded, as they did not present methods for the assessment of patient adherence. Study protocols were excluded due to lack of information on how adherence impacted on outcome.

Duplicate records were excluded, and the titles and abstracts of the remaining citations were independently screened by two reviewers (ZT, DY, or GD) using a standardised online screening pro forma, with an additional 5% screened at random by a third investigator (JH) to validate the screening process. The reference lists of retrieved articles and identified reviews were manually searched to identify additional potentially relevant studies. Full text articles were obtained for all potentially eligible records, and discrepancies were resolved by discussion (ZT, JMB, and JH).

### Selection of papers

To specifically focus on methods for assessing adherence, only papers including mention of ‘adherence’ in the title or abstract were included in the review.

RCTs, observational studies (prospective and retrospective), and case studies/series were eligible for inclusion if they assessed adherence to a removable orthosis in adult patients aged 18 and older following an acute injury or surgery. For the purposes of the review, the removable orthosis was required to fulfil the following criteria: (1) removable by the patient, (2) applied to a part of the appendicular skeleton, (3) immobilising or partially immobilising, e.g. allowing movement in one plane of motion, or allowing restricted movement for rehabilitation exercises, and (4) used following surgery or trauma. Studies on patients requiring orthoses for chronic conditions such as rheumatoid arthritis were excluded to focus the review on adherence in acute conditions.

Papers were screened for inclusion independently by two reviewers (ZT, DY, or GD) using a standardised inclusion criteria pro forma. Uncertainties that remained after full text review were resolved by discussion with the study team (JH, SP, JMB).

### Data extraction

#### Study demographics

Data were extracted using a standardised data extraction pro forma developed by the study team (ZT and JMB). Data extracted included (1) study design (RCTs, observational studies, and case series); (2) retrospective or prospective accrual of data; (3) study population and sample size; (4) condition evaluated; (5) details of the orthoses used; (6) duration of immobilisation.

#### Adherence measures

Included studies were further evaluated by considering details of the methods used for assessing adherence. These were classified as (1) questionnaire based, (2) diary based, (3) interview/consultation based, or (4) not stated (if no additional details were given). Details including instructions given regarding completion, timing of completion, the mode of completion (self-report, health professional administered, not stated), and frequency of assessment were extracted. For questionnaire-based studies, further information regarding the number and content of items in each instrument, the aspects of adherence assessed, and any scoring system used was also extracted.

The measures used were critically appraised to determine validity and reliability. This included details of whether the instruments used were validated or had been used in previous studies, and the risk of response bias. The risk of response bias in each assessment was evaluated as (1) high if the instrument/interview was conducted by the clinician involved in the patient’s care, (2) medium if the questionnaire was patient-reported but administered directly by a member of the clinical team, (3) low if the instrument was a self-administered questionnaire or consultation with a health professional unrelated to the study team, and (4) indeterminate if there were insufficient details given.

Finally, any details were extracted regarding whether the studies included any report of the association between adherence and outcome. As the aim of this review was to explore methods for assessing adherence, risk of bias within the individual studies themselves was not assessed.

Data extraction was performed by two reviewers (ZT, GD) with a third reviewer (JH) independently extracting approximately 30% for data validation purposes. Where there was uncertainty, this was discussed with JMB.

### Data analysis

Data were tabulated and details of assessment methods compared.

## Results

### Literature search and study selection

**Fig. 1:**
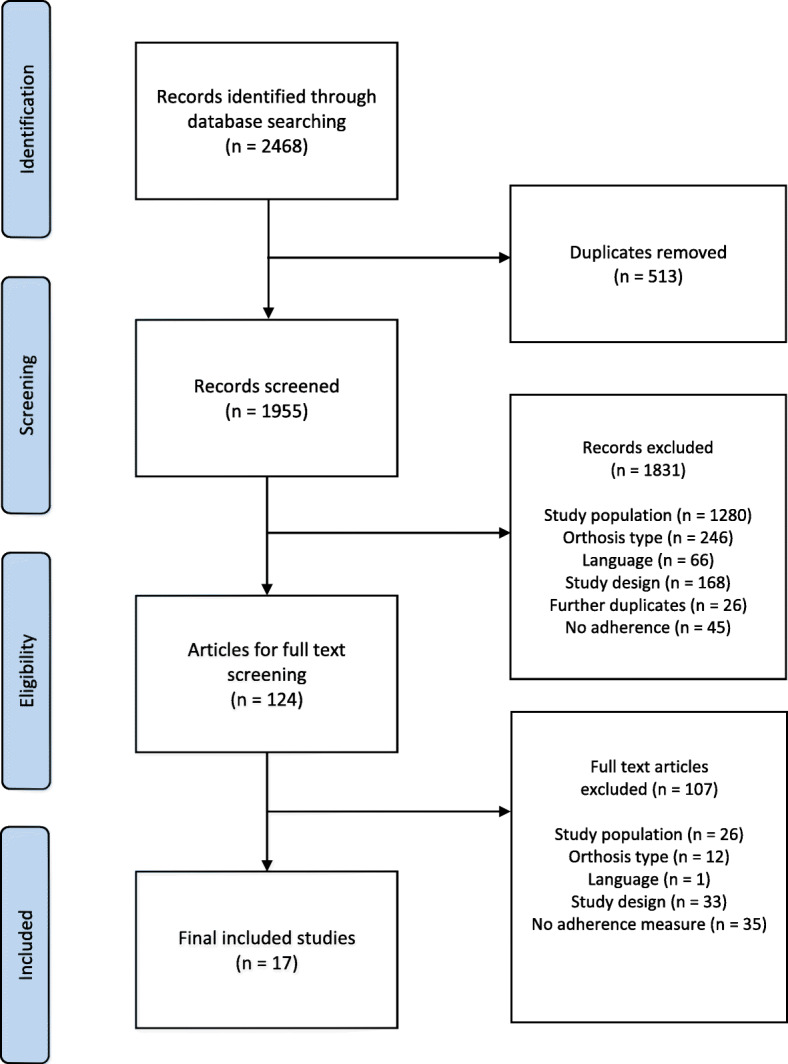
Preferred Reporting Items for Systematic Reviews and Meta-Analyses (PRISMA) flow diagram of included studies

From 1955 citations, 124 articles were obtained for full text screen and 14 systematic reviews for reference list screening (Fig. 1). The interrater reliability for title and abstract screening was 97%. Only 3% had discrepancies, which were resolved by full text screening. Some 17 studies met the inclusion criteria and were included in the analysis.

### Study design

The 17 studies included five RCTs [11, 19–22], eight prospective cohort studies [9, 12, 13, 23–27], two retrospective cohort studies [10, 28], and two individual patient case reports [29, 30]. Seven (41%) studies were single centre; 8 (47%) were undertaken in North America. The median sample size was 64 (range 1–188) (Table 1).

**Table 1:** Summary of included studies

	Number of studies
**Study design**	
RCT	5
Cohort	10
Case series	2
**Data collection**	
Prospective	13
Retrospective	4
**Number of centres**	
Single-centre	7
Multi-centre	6
Unclear	4
**Country**	
UK	2
Europe	3
North America	8
Asia	3
Australasia	1
**Sample size**	
Median	64
Range	1–188
**Anatomical location**	
Shoulder	5
Hand/wrist	8
Knee	2
Foot/ankle	2

The study populations included orthoses applied to the upper (hand [10–13, 25, 29], forearm [24, 28], shoulder [9, 19, 20, 22, 27]) and lower (knee [21, 26], ankle [23], foot [30]) limb. Details of the studies included in the analysis are provided in Table 2.

**Table 2:** Details of included studies

Author + year	Study design	No. of centres	Country	Sample size (no. of patients)	Medical condition	Type of orthosis	Instructions for daily use	Method of instruction	Duration of immobilisation	Adherence measure(s) used	Risk of bias in assessment of adherence
Cuff 2012 [9]	Prospective cohort	Unclear	USA	92	Post-op rotator cuff repair	Shoulder immobiliser	Removal only for exercise, bathing, dressing	Verbal	6 weeks	Single-item questionnaire + recorded therapy attendance	Low (objective assessment)
Groth 1994 [10]	Retrospective cohort	Multi-centre	USA	44	Acute mallet finger injury	Finger splint	Removal only for finger hygiene	Verbal	Between 6 and 8 weeks	Multi-item questionnaire + therapist observation + recorded therapy attendance	Indeterminate (insufficient information)
Guillodo 2011 [23]	Prospective cohort	Multi-centre	France	111	Sprained ankle	Aircast semi-rigid ankle brace	Not reported	Not reported	Between 5 and 6 weeks	Multi-item questionnaire	Low (independent assessor)
Itoi 2013 [19]	Randomised controlled trial	Multi-centre	Japan	109	Traumatic anterior shoulder dislocation	Shoulder immobiliser	Removal only for bathing	Verbal	3 weeks	Unclear	Indeterminate (insufficient information)
Liavaag 2011 [20]	Randomised controlled trial	Multi-centre	Norway	188	Traumatic anterior shoulder dislocation	Shoulder immobiliser	Removal only for sleeping, bathing, dressing	Verbal + written	3 weeks	Home diary	Low (independent patient report)
McGrath 2008 [24]	Prospective cohort	Unclear	USA	47	Wrist stiffness post-trauma/surgery	Adjustable wrist brace	Worn intermittently dependent on progress	Verbal	Dependent on progress (mean 10 weeks)	Discussion with patient	Indeterminate (insufficient information)
Midgley 2011 [25]	Prospective cohort	Single-centre	UK	50	Metacarpal fracture	Hand splint	Not reported	Written	4 weeks	Multi-item questionnaire	Low (independent assessor)
O’Brien 2011 [11]	Randomised controlled trial	Multi-centre	Australia	64	Acute mallet finger injury	Finger splint	Removal only for finger hygiene	Written	8 weeks	Home diary + therapist observation + recorded therapy attendance	Low (independent patient report)
Rives 1992 [13]	Prospective cohort	Unclear	USA	23	Surgically managed proximal interphalangeal joint (PIPJ) contraction	Finger splint	Worn continuously	Verbal	6 months	Discussion with patient	High (patient + clinician discussion)
Rankin 2000 [26]	Prospective cohort	Unclear	Canada	77	Post-op anterior cruciate ligament (ACL) reconstruction	Knee brace	Worn during high-risk sports	Not reported	6–18 months	Multi-item questionnaire	Low (postal questionnaire)
Roh 2016 [12]	Prospective cohort	Single-centre	South Korea	72	Acute mallet finger injury	Finger splint	Removal only for finger hygiene	Written	7 weeks	Single-item questionnaire	Low (independent assessor)
Sandford 2008 [28]	Retrospective cohort	Single-centre	UK	80	Post-op flexor/extensor tendon repair	Long forearm splint	Worn continuously	Not reported	4 weeks	Multi-item questionnaire	Low (independent patient report)
Silverio 2014 [27]	Prospective cohort	Single-centre	USA	50	Post-op rotator cuff repair	Shoulder immobiliser	Removal only for exercise, bathing, dressing	Verbal	6 weeks	Multi-item questionnaire	Medium (questionnaire given by clinician at follow-up)
Swirtun 2005 [21]	Randomised controlled trial	Single-centre	Sweden	95	ACL rupture (conservative management)	Knee brace	Worn during all daytime activities	Verbal	12 weeks	Single-item questionnaire	Indeterminate (insufficient information)
Whelan 2014 [22]	Randomised controlled trial	Multi-centre	Canada	60	Traumatic anterior shoulder dislocation	Shoulder immobiliser	Removal only for exercise, bathing, dressing	Written	4 weeks	Home diary + multi-item questionnaire	Low (independent patient report)
Wollstein 2012 [29]	Case report	Single-centre	USA	1	PIPJ contracture	Finger splint	Not reported	Not reported	12 weeks	Home diary	Indeterminate (insufficient information)
Yang 2011 [30]	Case report	Single-centre	Taiwan	1	Lisfranc injury	Foot brace	Not reported	Not reported	Unclear	Discussion with patient	High (patient + clinician discussion)

### Adherence measures

The methods used to assess adherence varied widely between studies. Ten (59%) used questionnaires, four (24%) used patient-reported diaries completed at home, three (18%) involved a patient discussion, and in one study the method used was unclear. Other stated methods for assessing adherence included recorded therapy attendance (*n* = 3, 18%) and therapist observation of study participants (*n* = 2, 12%). Most studies (*n* = 13/17, 76%) relied on a single measure of adherence, whilst four (two RCTs [11, 22] and two cohort studies [9, 10]) assessed adherence using a combination of measures. Studies using more than one method did not differ greatly from the remaining studies in terms of sample size or geographical location, but three of the four studies were multi-centre, and all involved orthoses applied to the upper limb. Eight studies (47%) used methods for assessing adherence that were at low risk of bias, as they were completed independently of the clinicians delivering patient care. The remaining methods were either at high risk of bias or insufficient detail was provided for an assessment to be made. None of the included studies used an adherence measure that had been validated for use in this patient group. None of the studies reported patient or staff feedback regarding the adherence methods used.

#### Patient questionnaires

Ten studies used patient questionnaires to assess adherence. The majority utilised multi-item questionnaires, but three studies used single-item measures.

##### Single-item questionnaires

Of the three single-item measures, two were patient self-reported and one used clinician assessment. All measures were study-specific. There was a lack of consistency in the target behaviour recorded and in the way the adherence scores were determined. Whilst two studies focussed on total orthosis usage, the third recorded instances when the orthosis was removed. The method of deriving an adherence score varied from a percentage of orthosis use, to a 3-point scale of compliance, to a dichotomous (compliant vs non-compliant) method (Table 3). None of the instruments was reported to be validated, and only one study [12] used a referenced adherence score. This study used a modified version of the Groth et al. [10] 3-point scoring system that divides patients into compliant, secondary compliant, and non-compliant, although there is very limited information on the development of this tool, and the validity of the classification system is limited.

**Table 3:** Single-item questionnaire information

Study name	Assessor	Method of questionnaire administration	Validated tool?	Timing of assessment	Description of measure		Adherence score
					Target behaviour	Response type	
Swirtun 2005 [21]	Patient	‘Paper’ no other details provided	No	Weeks 8 & 12 post-injury	Daily usage of brace	Percentage of daily activities brace used (ordinal): 0–25%, 26–50%, 51–75%, 76–99%, 100%	Percentage in each group
Cuff 2012 [9]	Clinician	Unclear	No	Days 1, 2, 3 (home visit) Clinic at 1, 3, 6 weeks	Wearing brace	Dichotomous: Yes/No	Non-compliant: 1+ non-compliant event recorded
Roh 2016 [12]	Patient	Unclear	No	Week 7 post-injury (at splint removal)	Splint removal	Ordinal: 3 = never removed (or only with extreme care), 2 = accidentally dislodged or loose but instantly replaced, 1 = not worn properly or removed several times	3: Compliant 2: Secondary compliant 1: Non-compliant

##### Multi-item questionnaires

Multi-item questionnaires were used in almost half of the included studies (7/17; 41%). These were patient self-report questionnaires completed by patients in person in clinic (*n* = 2) or distributed by post (*n* = 1), and questionnaires administered by telephone (*n* = 2). The mode of questionnaire administration was unclear in the remaining two studies (Table 4). Three studies [23, 27, 28] included a copy of their study questionnaire in the final publication. As with the single-item measures, there was a lack of consistency in the target behaviours assessed, the timing of assessment, and the use of ‘adherence scores’. Some tools focussed on total orthosis use, whilst others recorded specific instances of orthosis removal (Table 4). Three of the seven studies using multi-item measures formed a combined adherence score used for analysis, whilst the others considered each of the items separately. There was also significant variation in the timing of the adherence assessment, ranging from 4 weeks to 36 months.

**Table 4:** Multi-item questionnaire information

Study name	Assessor	Method of questionnaire administration	Validated tool?	Timing of assessment	Description of measure			Adherence score	
					No. of items	Target behaviour	Response type	Combined score?	Description
Groth 1994 [10]	Patient	Unclear	Unclear	Unclear	2	1. Use of splint as prescribed 2. Adherence to exercise programme	Dichotomous: Yes/No	Yes	Adherent = Yes to both
Guillodo 2011 [23]	Patient	Telephone call	No	60–90 days post-injury	2	1. Use of brace 2. Length of use (total days)	1. Dichotomous Yes/No 2. Continuous: no. of days	No	Reported individually
Midgley 2011 [25]	Patient	Telephone call	No	Min 10 weeks post-injury	2	1. Compliance with splint use (subjective) 2. Length of use (total weeks)	1. Dichotomous Yes/No 2. Continuous: no. of weeks	No	Reported individually
Rankin 2000 [26]	Patient	Postal questionnaire	Pre-tested, not validated	12–36 months post-reconstruction	16	1. Compliance with splint use during different sports 2. Compliance with home exercise programme	Continuous: visual analogue scale (100-mm line)	No	Reported individually
Sandford 2008 [28]	Patient	Paper questionnaire	No	Clinic appt 4 w post-surgery	4	1. Has splint been removed? 2. Frequency of removal 3. Duration of removal 4. Reasons for removal	1. Dichotomous: Yes/No 2. Ordinal: never, once, 2–6 times, daily 3. Ordinal: < 1 h, > 1 h 4. Descriptive	No	Reported individually
Silverio 2014 [27]	Patient	Paper questionnaire	Not in this population	Clinic appt 6 w post-surgery	4	1. Daily hours without sling 2. Days per week without sling 3. Why was sling removed? 4. Subjective adherence	1. Continuous 2. Continuous 3. Nominal 4. Scale of 1–10	Yes	Adherence (%) = 100 x [(hours of sling use/ 24 × 0.5) + (% activities performed with sling on × 0.25) + (self-ranked adherence/ 10 × 0.25)]
Whelan 2014 [22]	Patient	Unclear	No	After 4 w of immobilisation	2	1. Was brace used full time? 2. Was brace used for whole (4-w) period?	Not reported	Yes	Compliant = full time use for at least 75% (3 w out of 4) of immobilisation period

*h* hours, *w* weeks

None of the multi-item questionnaires identified had been validated for use in this population. Silverio and Cheung [27] adapted a previously published medical adherence measure [31] originally developed for use in a paediatric population. No information, however, was provided on how the adaptations (e.g. question wording, response options, scoring system) were made, or whether the adapted version had been validated in an adult population.

Another measure used by Rankin et al. [26] was reported as being developed by four surgeons and four physical therapists and subsequently pre-tested on six patients before use. However, this pre-testing only assessed simplicity and ease of completion and did not provide any additional data on the measurement properties.

#### Home diaries

A total of four studies used home diaries to assess adherence. All home diaries were patient-completed, but there was no consistency in the frequency of information recording, items assessed, and how individual patient adherence was scored (Table 5). Some diaries involved daily recording of hours of orthosis usage, whilst others focussed only on instances of orthosis removal. One diary involved a fortnightly recall of average daily hours of orthosis use in the preceding 2 weeks. These differences are summarised in Table 5.

**Table 5:** Home diary information

Study name	Frequency of information recording	Instructions for adherence information recording	What information was recorded	Adherence score
Liavaag 2011 [20]	Daily	Total duration of use (days) + daily duration of immobilisation (hours) 1. No use 2. < 8 h 3. 8–16 h 4. > 16 h	Total no. of days used + hours per day: 0 h, < 8 h, 8–16 h, > 16 h	Compliant = > 16 h for 20 + days (otherwise non-compliant)
O’Brien 2011 [11]	As needed	Any instances of splint removal, modification, dislodgement	Time/date of incident + reason for incident	• Compliant: never removed (or only with extreme care) • secondary compliant: splint dislodged/loose but instantly replaced • Non-compliant: splint not worn properly/removed multiple times
Whelan 2014 [22]	As needed	Brace/sling usage + attendance at physical therapy	Unclear	Unclear
Wollstein 2012 [29]	Fortnightly	Average daily splint wear (hours)	No. of hours	No adherence score

*h* hours

One study [11] used a modified version of the Groth et al. [10] 3-point scoring system, again without evidence of validation.

#### Interview/consultation-based methods

Three studies used unstructured discussions between clinician and patient to assess adherence. Whilst one reported relying on the patient volunteering a compliance of less than 50% to be deemed as non-compliant, the actual assessment for the other studies was much less clear. They did not provide any detailed information regarding the nature of these discussions and how exactly the adherence was assessed. Given the direct involvement of the responsible clinician in assessing adherence, each of these studies was at high risk of bias.

### Association between adherence and outcome

Just over half of the included studies (*n* = 9) considered the association between adherence data and study outcomes. The nature of these associations varied widely. Some studies provided a narrative description of associations only, whereas others provided quantitative statistical comparisons of outcomes or correlations between adherence and outcome. Much of this variety was related to the wide variation in final reporting of adherence data. There was also significant heterogeneity in the results of the reported association. Whilst some papers reported an improvement in clinical outcome with improved adherence, others did not find any difference. The reporting of associations between adherence and outcome is summarised in Table 6.

**Table 6:** information of association between adherence and outcome

Study name	Type of association	What was reported?	Were outcomes improved in compliant patients?
Cuff 2012 [9]	Statistical quantitative analysis	Comparison of outcomes between compliance groups	Yes
Groth 1994 [10]	Statistical quantitative analysis	Comparison of outcomes between compliance groups	Yes
Guillodo 2011 [23]	Statistical quantitative analysis	Correlation between length of brace use + subjective assessment of recovery	No
Midgley 2011 [25]	Narrative analysis	States no difference in outcomes with length of orthosis use	No
O’Brien 2011 [11]	Statistical quantitative analysis	Correlation between compliance + clinical outcome	Mixed
Rives 1992 [13]	Descriptive quantitative analysis	Outcomes for each group without statistical comparison	Yes
Roh 2016 [12]	Statistical quantitative analysis	Multivariate regression analysis of adherence as a predictor of outcome	Yes
Sandford 2008 [28]	Narrative analysis	Recorded individual compliance of patients with poor outcome	Inconclusive
Silverio 2014 [27]	Statistical quantitative analysis	Correlation between compliance + clinical outcome	No

## Discussion

This is, to our knowledge, the first systematic review to identify, summarise, and critically appraise methods for assessing adherence to removable orthoses used after surgery or trauma to the appendicular skeleton. In the 17 included studies, patient self-report questionnaires were the most commonly used method of measuring adherence. These were commonly multi-item questionnaires, but single-item questionnaires, home diaries, and informal history taking were also used. Overall, there was a lack of consistency in the target behaviour assessed, the timing and frequency of assessment across studies, and whether adherence overall was scored. None of the instruments was validated in the study population. Approximately half of the included studies used methods of assessing adherence that minimised response bias (e.g. postal questionnaires), but the remaining studies used methods such as direct questioning which may have impacted on participants’ responses or did not provide sufficient details (e.g. how and by whom the measure was administered) for the risk of bias in the assessment to be assessed. The lack of consistency in the way that adherence is measured and reported was unanticipated, given the importance of the adherence in orthosis use and its potential impact on outcomes. A validated approach for assessing adherence to removable orthoses is recommended to be used in studies evaluating their effectiveness and will be vital in any future splinting trial. To our knowledge, no such measures exist, and work to develop suitable tools is therefore required. Such measures may need to include reasons for non-compliance.

The challenges of measuring adherence are not unique to orthosis use. Systematic reviews have previously evaluated methods used to assess adherence to medication usage [32], home-based rehabilitation [17], and prescribed exercise [33]. The most widely studied field is that of adherence to medication, with a wide range of tools being designed and validated over the past four decades, and yet none of them can be considered the gold standard [32]. The ideal adherence measure should be economically viable, user friendly, reliable, sensitive, and practical [34, 35]. As no single measure has been found to meet all of these standards, a combination of methods is often recommended to account for the limitations of individual tools [35].

The vast majority of the measurement tools identified in this study were self-reported measures. These provide a low-cost, flexible, practical, and easily implemented measure of adherence. Given the unsupervised nature of recommended orthosis use, these measures provide a straightforward method of collecting otherwise difficult-to-obtain adherence data. However, they do have significant limitations. It has long been known that patients are often unwilling to admit non-adherence [4], and measures may overestimate the true value. Self-reported measures are vulnerable to social desirability bias, with respondents providing answers they feel will please their healthcare professionals [36]. This is a particular problem if the measure is administered by a clinician. Self-report questionnaires may reduce this, but recall bias should be considered if the adherence is assessed several weeks after the injury or intervention. These factors, combined with the study-specific non-validated nature of the measures used, call into question the reliability of the adherence data reported in these studies. These problems have also been shown in other settings. Indeed, Shi et al. [37] reviewed 41 studies assessing the agreement between self-reported measures of medication adherence and electronic drug monitoring and demonstrated a significantly lower correspondence between self-report and electronically monitored adherence when non-named, non-standardised self-reported measures were used.

The widespread use of non-standardised study-specific instruments also limits the comparability of study findings and the ability to pool data in meta-analyses, as highlighted in previous systematic reviews [38, 39]. Although improving adherence to recommended therapies is widely considered to improve outcomes [40, 41], the nature of this relationship is not always straightforward. The complexity of this association may partly be explained by the poor-quality measurement of adherence data that currently exists. There is therefore a need for a validated standardised measure of adherence for use in this setting to improve the quality and comparability of future research in this field.

There are several limitations to our systematic review. Firstly, the review was restricted to papers including the term adherence in the title or abstract. This approach may have excluded studies where relevant methods of assessing adherence were used but not reported in the abstract. The focus of this review, however, was specifically to evaluate methods of measuring adherence, and most of the studies providing a detailed description of this process measure commented on this in the abstract. Restricting the review to use of orthoses in acute settings is another potential weakness. However, the psychological underpinnings of adhering to short-term and long-term treatments with a removable orthosis in these circumstances are likely very different; thus, they potentially require different monitoring approaches for optimisation [42]. A similar rationale can be applied to restricting the review to adults. Overall, however, this study has provided a snapshot of current methods for assessing adherence to orthosis use, and has demonstrated the need for improvement.

Given the importance of adherence to orthosis use in determining outcomes in research and clinical practice, there is a need to improve the quality and consistency of the measurement and reporting of this key outcome. This is particularly relevant to ongoing trials such as Osteoarthritis Thumb Therapy (OTTER) II (ISRCTN 54744256) where the clinical and cost-effectiveness of a splint is being compared to a placebo splint and optimal self-management for the treatment of symptomatic thumb osteoarthritis [43]. A valid, robust, and reliable measure is therefore required that is acceptable to patients and can be used across all studies. This may be challenging, given the variation in types of orthoses used, recommendations regarding duration of use, and other clinical variables, and any measure would need to be sufficiently flexible to meet these needs. However, there are generic components of adherence to orthoses that could be included. We recommend that work with key stakeholders including surgeons, therapists, and patients and their carers is needed to ensure that the measure is appropriate and can be effectively used across different conditions. Key design features will include ease of use and digital solutions, as electronic patient-reported outcomes have been shown to be useful in similar patient groups [44].

However, more objective measures of adherence are also likely to be needed [45], and technological advances such as the development of sensors that can be placed in any orthosis may offer an ideal solution by allowing accurate and detailed information to be reliably collected with minimal burden to patients [46, 47]. Such sensors are amenable for use in a trial setting and are also cheap and relatively ‘low tech’, making it possible to integrate them into routine practice.

This review has highlighted the need to improve methods for assessing patient adherence to orthoses following surgery, but development of appropriate tools will take time, and simple recommendations to improve the quality and value of upcoming and ongoing work can be proposed based on the findings of this review. Firstly, it is vital that trialists select measures that capture the most important aspects of adherence for their study and aim to collect data in a way that represents a minimal burden to patients whilst minimising bias. Self-completed patient questionnaires may represent the best option, and it may be possible to validate instruments already in use.

Assessing adherence may also be more important at different stages in evaluation. Robust assessment would be particularly important in explanatory trials when determining if an orthosis was an appropriate treatment in an ideal settling with a compliant patient, but it may be less important in a large-scale pragmatic study when the aim would be to evaluate effectiveness in the ‘real world’. Similarly, reasons for non-compliance to orthosis use would have particular value in the pilot and feasibility stages of a trial. It may be that the instructions for use or the orthosis itself could be modified based on patient feedback so that the intervention would be more acceptable to patients in the main study and thus more likely to be used. At present, the best recommendations for trialists involved in studies using orthosis are that details of any methods used for assessing adherence be well described (e.g. included as a study additional file) and transparently reported so that readers can judge for themselves the methods used and the results. Transparent reporting of any statistical analysis used for assessing the impact of adherence on outcomes is similarly vital, including details of any assumptions made and any limitations of the measures used. Researchers should take care when interpreting their findings to take account of the limitations of the measures of adherence currently used and to acknowledge these issues when discussing the implications of the results for both further research and clinical practice.

## Conclusions

Measurement and reporting of adherence to orthosis use is currently inconsistent, resulting in problems with interpretation of relevant literature and impacting on understanding of the efficacy of orthoses prescribed in the acute setting following injury or surgery. Further research is required to develop measurement tools that provide a rigorous and reproducible assessment of adherence in this acute population.

## Supplementary information

**Additional file 1.** Search strategy MEDLINE (Ovid).

## Data Availability

All data generated or analysed during this study are included in this published article and its additional file.
