# Supplementary material for: Methods for assessment of patient adherence to removable orthoses used after surgery or trauma to the appendicular skeleton: a systematic review
Source: Trials. 2020 Jun 8;21:507. doi: 10.1186/s13063-020-04456-2 (PMC7278128; doi:10.1186/s13063-020-04456-2)
Supplement: Supplementary file 1 — Additional file 1. Search strategy MEDLINE (Ovid). [file 13063_2020_4456_MOESM1_ESM.docx]

**Additional file 1: Search Strategy MEDLINE (OVID)**

Ovid MEDLINE(R) In-Process & Other Non-Indexed Citations and Ovid MEDLINE(R)

1 patient compliance/

2 "patient compliance".tw.

3 "patient adherence".tw.

4 "therapy adherence".tw.

5 compliance.ti,ab.

6 adherence.ti,ab.

7 ("self care" or "self-care" or "self report*" or "self-report*" or "information leaflet*" or "treatment plan*").ti.

8 or/1-7

9 splints/

10 braces/

11 orthopedic equipment/

12 orthotic devices/

13 splint*1.ti,ab.

14 brace*1.ti,ab.

15 orthos#s.ti,ab.

16 orthotic.ti,ab.

17 hand injuries/

18 leg injuries/

19 arm injuries/

20 finger injuries/

21 or/9-20

22 8 and 21

23 back injuries/ or back pain/ or abdominal injuries/ or neck injuries/ or spinal cord injuries/ or thoracic injuries/ or tooth injuries/ or orthodontics/ or skull fractures/ or mandibular fractures/ or mandible/ or temporomandibular joint disorders/ or facial bones/ or scoliosis/ or thoracic vertebrae/ (180925)

24 22 not 23

25 limit 24 to yr="1990 -Current"
